# Subclinical Cardiovascular System Changes in Obese Patients with Juvenile Idiopathic Arthritis

**DOI:** 10.1155/2013/436702

**Published:** 2013-03-11

**Authors:** Barbara Głowińska-Olszewska, Artur Bossowski, Elżbieta Dobreńko, Andrzej Hryniewicz, Jerzy Konstantynowicz, Robert Milewski, Włodzimierz Łuczyński, Janina Piotrowska-Jastrzębska, Otylia Kowal-Bielecka

**Affiliations:** ^1^Department of Pediatrics, Endocrinology, Diabetology with Cardiology Division, Medical University of Bialystok, Waszyngtona 17 Street, 15-274 Białystok, Poland; ^2^Department of Pediatrics and Developmental Disorders, Medical University of Bialystok, Waszyngtona 17 Street, 15-274 Białystok, Poland; ^3^Department of Statistics and Medical Informatics, Medical University of Bialystok, Szpitalna 37 Street, 15-295 Białystok, Poland; ^4^Department of Rheumatology and Internal Medicine, Medical University of Bialystok, M. Skłodowskiej-Curie 24A Street, 15-276 Białystok, Poland

## Abstract

*Objective*. We aimed to determine the prevalence of excess body mass in juvenile idiopathic arthritis (JIA) children and to investigate the influence of obesity into the early, subclinical changes in cardiovascular system in these patients. *Methods*. Fifty-eight JIA patients, aged median 13 years, were compared to 36 healthy controls. Traditional cardiovascular risk factors and inflammatory markers (hsCRP, IL-6, TNF**α**, adiponectin) were studied together with IMT (intima-media thickness), FMD (flow mediated dilation), and LVMi (left ventricle mass index) as surrogate markers of subclinical atherosclerosis. *Results*. Thirteen JIA children (22%) were obese and had increased systolic blood pressure, cholesterol, triglycerides, insulin, HOMA, hsCRP, and IL-6 compared to nonobese JIA and controls. FMD was decreased compared to nonobese JIA and controls, whereas IMT and LVMi were increased. In multivariate regression analysis, TNF**α**, SDS-BMI, and systolic blood pressure were independent predictors of early CV changes in JIA. *Conclusions*. Coincident obesity is common in JIA children and is associated with insulin resistance, dyslipidemia, and increased levels of inflammatory markers leading to early changes in cardiovascular system. Thus, medical care of children with JIA should include strategies preventing cardiovascular disease by maintenance of adequate body weight.

## 1. Introduction

The atherosclerotic process begins in childhood, with the progression clearly shown to be mediated by the presence of identified risk factors. Studies in children and young adults demonstrate that the extent of the atherosclerotic vascular change is associated with both number of risk factors and their intensity [1, 2]. For most children, the degree of vascular involvement is minor and the rate of progression is slow. By contrast, certain chronic pediatric conditions are associated with accelerated atherosclerosis, including clinical coronary events occurring in childhood, with homozygous hypercholesterolemia being the classic example. Recently, chronic inflammatory diseases, including juvenile idiopathic arthritis (JIA), have been considered pediatric conditions producing high risk for a premature cardiovascular disease (CVD) [3]. 

Adults with chronic inflammatory disease, specifically patients with rheumatoid arthritis (RA), experience cardiovascular events at a significantly greater incidence than age-matched controls, and cardiovascular disease is reported the major cause of mortality in RA [4, 5]. The prevalence of traditional cardiovascular (CV) risk factors in RA has been widely investigated, and despite some inconsistent results, comparable prevalence has been noted among RA patients and in the general population [6]. Traditional factors related to CV or RA independently predict CV diseases, and the risk increases with the number of both types of parameters [7]. The close associations between obesity, obesity-related adipokines, inflammation, and CVD render the study of this risk factor in RA highly significant [8]. Results from studies on adults with RA have shown that overweight and obesity significantly increase their 10-year risk of CVD event [9].

There are very scarce published data on subclinical atherosclerosis in patients with JIA, reviewed in [10]. Recent studies showed increased IMT in JIA children or impaired endothelial function in these patients [11, 12]. However, children with traditional atherosclerosis risk factors were not included in those studies. There is evidence that obesity in childhood is a growing phenomenon almost all over the world and is predictive for adulthood obesity and cardiovascular disease [13, 14]. Based on trends towards obesity observed among young populations, a strong likelihood arises that increasing proportion of patients with JIA may also demonstrate excess body weight. To our best knowledge, no study has so far assessed subclinical changes in cardiovascular system in relation to overweight and/or obesity in JIA. 

At present, several noninvasive imaging techniques used in children offer an opportunity to study the relationship of surrogate markers to the development of atherosclerosis. The use of these techniques may help to identify high-risk individuals in preclinical phase who may benefit from active therapy to prevent clinical disease. Endothelial function, that is, the vasodilator response to increased blood flow (flow mediated vasodilatation (FMD)), the analysis of carotid artery intima-media thickness (IMT), and echocardiographic assessment of left ventricle mass and/or hypertrophy (LVM), can now be accomplished using high-resolution ultrasound [15].

Therefore, the aims of the study were, firstly, to investigate the prevalence of overweight and obesity in children with juvenile idiopathic arthritis and, secondly, to examine possible associations between excess body weight and traditional cardiovascular risk factors together with selected inflammatory markers and early changes in cardiovascular system, which may predispose this group of patients into early clinically symptomatic atherosclerosis. We hypothesized that overweight or obese children and adolescents with JIA had increased surrogate markers of subclinical atherosclerosis in comparison to nonobese JIA patients.

## 2. Material and Methods

### 2.1. Study Participants

We recruited 58 consecutive JIA children, 32 (55%) girls, aged 7–18 (median 13 years), and diagnosed with juvenile idiopathic arthritis (according to ILAR criteria) for at least one year; they were followed at the tertiary academic center, Medical University of Bialystok, Poland. Oligoarticular, polyarticular, and systemic types of JIA were reported in 28 (48%), 26 (45%), and 4 (7%) patients respectively. Children were divided into clinically active (30–52%) and inactive (28–48%) based on current practice recommendations [16]. The recruitment for the study group, all clinical examinations, and qualification to the groups were performed by an experienced pediatric rheumatologist (ED). The control group included 36 normal weight children, 22 (61%) girls and aged 8–18 (median 13.4 years) with negative family history of CVD and absence of systemic inflammatory disease based on physical and laboratory examination. These reference children were recruited from patients admitted to our hospital due to minor cardiologic problems who were otherwise healthy. The inclusion criteria for the control group was BMI below the 85th percentile. 

All children underwent physical examination; height and weight were taken in a standard way using the Harpenden stadiometer and digital scale (Seca, Germany). Body mass index (BMI) was calculated with a standard formula. Overweight was determined when the BMI (kg/m^2^) exceeded the 85th per centile whereas obesity as BMI exceeding the 95th per centile according to national growth references [17]. Because the BMI is not normally distributed in childhood, we used the least mean square method [18], which normalizes the BMI skewed distribution and expresses BMI as an standard deviation score (SDS-BMI). Systolic (SBP) and diastolic (DBP) blood pressures were measured twice at the right arm after a 10-minute rest using calibrated sphygmomanometer with appropriate cuff size and were averaged. The pubertal development was determined by a physician according to the Tanner classification and participants were categorized into prepubertal (Tanner's stage 1) or pubertal (stages 2–5).

### 2.2. Laboratory Investigations

A blood sample of 10 mL was taken from the left cubital vein, after an overnight (8–12 hr) fast. To assess inflammatory markers, serum samples were collected, frozen, and stored at the temperature of −80°C until analyses were performed. The concentrations of adiponectin, IL-6, and TNF*α* were determined immunoenzymatically using commercially available ELISA kits (Parameter Human Immunoassays, R&D Systems, Inc., Minneapolis, USA) with the use of ELx 800 Automated Microplate Reader, Bio-Tek Instruments, Vermont, USA. hsCRP was determined with use of immunoturbidimetric method (Tina-quant hsCRP (Latex) HS, Roche; Hitachi 912, La Roche, Japan). Concentrations of lipid, glucose, and insulin parameters were determined by standard enzymatic methods (Hitachi 912, La Roche, Japan). LDL concentration was assessed by the Friedewald equation. The homeostasis model was used to assess insulin resistance (HOMA IR) derived from the following formula: insulin resistance (HOMA IR) = (fasting insulin (mU/ml) × fasting glucose (mmol/l))/22.5.

#### 2.2.1. Ultrasound Measurements

The procedure was conducted between 8.00 and 10.00 AM after a fasting period of 8–12 hours. Examinations of the brachial and carotid arteries were performed with Hewlett Packard Sonos 4500 apparatus, using a 7.5 MHz linear transducer. Ultrasound examination of the right brachial arteries was performed in longitudinal sections 2–10 cm above the elbow, according to guidelines [19]. The principle is to induce vasodilatation in the proximal (brachial) artery by postischemic (forearm) enhanced flow. All lumen diameter measurements were scanned at end diastole by use of the R-wave of the electrocardiogram. First scans were taken at rest and second scans during reactive hyperemia. Increased flow was induced by deflating a pneumatic tourniquet placed on the right forearm, inflated to the pressure about 50 mmHg above the patient's resting systolic blood pressure for 4.5 min. The postischemic scan was performed 45–120 seconds after cuff deflation. FMD was derived from the percentage change of the brachial artery diameter after ischemia of the forearm from baseline.

Measurements of intima-media thickness (IMT) in the common carotid arteries (right and left) were performed as previously described, with own modification [20, 21]. Measurements included end-diastolic (minimum diameter) IMT of the far walls (the distance from the leading edge of the first echogenic line to the leading edge of the second echogenic line), at the distance of more than 1 cm from the bifurcation. Analyses included the mean value of 6 measurements. 

All participants underwent a complete 2D echocardiogram with M-mode and Doppler study demonstrating structurally normal heart. Measurements of the left ventricle (LV) internal dimension, interventricular septal thickness, and posterior wall thickness were made during diastole according to practice guidelines of the American Society of Echocardiography. Left ventricle mass index (LVMi) was calculated by dividing LV mass by height in meters raised to the power of 2.7 to minimize the effect of age, gender, and body weight [22].

All the examinations were carried out and analyzed by one experienced pediatric vascular ultrasonographer (AH), who was blinded to the participants' cardiovascular risk factor status. The intraobserver variability was 2.5% for FMD and 3.2% for IMT (evaluated in a subset of patients, *n* = 20).

We obtained approval of the Ethical Committee in the Medical University of Bialystok. Both parents/legal guardians and children gave their written informed consent.

### 2.3. Statistical Analysis

All continuous variables were tested for normal distribution by the Kolmogorov-Smirnov test, with Lilliefors correction and Shapiro-Wilk tests. As most of the studied parameters were not normally distributed, descriptive statistics were calculated as median with the interquartile range: median (IQR). The Mann-Whitney *U* test was used to compare continuous variables, and *χ* ^2^-test with the Yates correction was used to compare categorical variables between two groups. ANOVA Kruskal-Wallis test with post hoc analysis for multiple comparisons was used to compare more than two groups. The correlations between studied variables were assessed using the Spearman correlation. In order to detect independent determinants of FMD, IMT, and LVMi, multiple linear regression analysis was performed. The independent association between each subclinical atherosclerosis marker and overweight/obesity reported as SDS-BMI was assessed in two regression models. Base model included traditional atherosclerosis risk factors: obesity, hypertension, dyslipidemia, and insulin resistance. Furthermore, the extended model additionally included major inflammatory mediators, that is, Il-6, TNF*α*, and hsCRP concentrations. Only variables for which the *P* value in correlation analysis was ≤0.05 were included in these models. All analyses were adjusted for age, pubertal status, and corticosteroid use. Statistical significance was determined at *P* < 0.05 level. All calculations were made using Statistica 8.0 StatSoft.

## 3. Results

Demographic characteristics and clinical data are shown in Table 1. Patients were similar to controls with respect to age, gender, and pubertal status; however, patients were shorter. Thirteen children with JIA (22%) fulfilled criteria for overweight or obesity, and they were separated as a subgroup named obese (OB) JIA patients and compared with nonobese JIA patients and controls in further analyses. 

### 3.1. Cardiovascular Risk Factors and Subclinical Atherosclerosis in the JIA and Control Groups

The JIA children demonstrated significantly increased SDS-BMI, systolic and diastolic blood pressure, hsCRP, IL-6, TNF*α*, IMT, and LVMi and decreased FMD compared to control group. Adiponectin level did not differ between the study groups. None of the JIA children had hypertension although the SBP and DBP were higher in this group (Table 2).

### 3.2. Differences between Obese JIA, Nonobese JIA, and Control Groups

**Figure 1:**
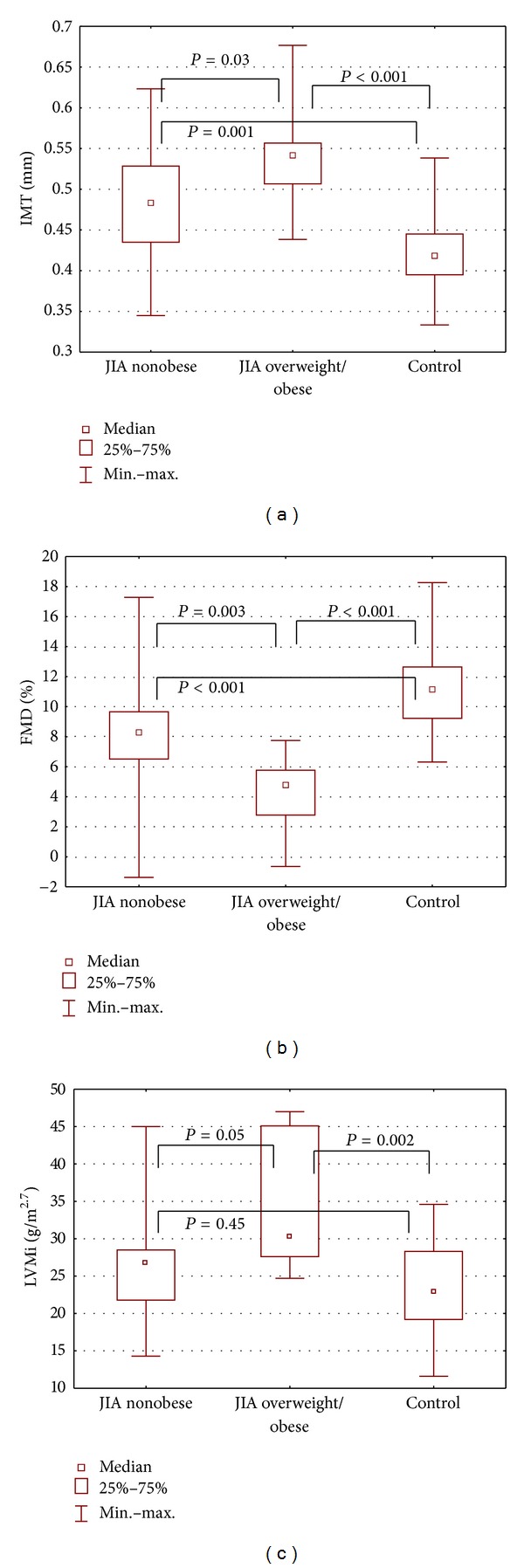
Subclinical changes in the cardiovascular system in 58 children and adolescents with juvenile chronic arthritis (JIA) divided depending on presence of excess body mass (nonobese JIA, overweight/obese JIA, controls). (a) Intima-media thickness (IMT), (b) endothelial function—flow mediated dilation (FMD), and (c) indexed left ventricle mass (LV mass indexed by height in meters raised to the power of 2.7 to minimize the effect of age, gender, and body weight).

Children with obesity (OB) had higher BMI and SDS-BMI compared to nonobese or controls (*P* < 0.001 for all post hoc comparisons). OB had significantly increased SBP compared to controls (*P* = 0.02), and the difference between nonobese and controls was no further significant. OB group had also significantly increased total cholesterol (*P* < 0.001 versus nonobese, *P* = 0.03 versus control), triglycerides (*P* = 0.04 versus nonobese), fasting insulin (*P* < 0.001 both versus nonobese and control), insulin resistance index HOMA (*P* = 0.002 both versus nonobese and controls), hsCRP (*P* = 0.01 versus nonobese, *P* = 0.002 versus control), and IL-6 (*P* = 0.01 versus nonobese, *P* < 0.001 versus control). TNF*α* was slightly higher in the OB group (Table 3). Surrogate markers of atherosclerosis in OB were as follows: FMD was decreased (*P* = 0.003 versus nonobese, *P* < 0.001 versus control), while IMT (*P* = 0.03 versus nonobese, *P* < 0.001 versus control) and LVMi (*P* = 0.002 versus control) were increased (Table 3, Figure 1). The differences between hsCRP, Il-6, TNF*α*, FMD, and IMT remained significant also between nonobese JIA and controls. Adiponectin level was similar in the groups divided regarding prevalence of obesity. Obese versus nonobese groups did not differ considering treatment methods: corticosteroids: 10 (76%) versus 32 (71%), *P* = 0.9; methotrexate: 5 (38%) versus 23 (51%), *P* = 0.6; biologic agents: 3 (23%) versus 11 (24%), *P* = 0.7, and DMARDs: 3 (23%) versus 6 (13%), *P* = 0.7. 

### 3.3. Differences between Clinically Active and Inactive JIA and between Types of the Disease and Controls

Clinically active disease was associated with higher SDS-BMI compared to controls (*P* = 0.009), whereas no differences were found between inactive JIA patients and controls. SBP was highest in inactive JIA patients (*P* = 0.04 versus control). Clinically active JIA patients had the highest values of hsCRP (*P* < 0.001 versus control) and Il-6 (*P* < 0.001 versus control), although not different from inactive JIA patients. FMD was the lowest (*P* < 0.001 versus control), and LVMi was significantly higher in clinically active JIA patients (*P* = 0.04 versus control). No differences were found in subclinical markers of atherosclerosis between active and inactive JIA (Table 4). Clinically active versus inactive patients differed significantly regarding treatment regimens: corticosteroid use: 27 (90%) versus 15 (53%), *P* = 0.005; methotrexate: 21 (70%) versus 7 (25%), *P* = 0.001; biologic agents: 11 (37%) versus 3 (10%), *P* = 0.04; and DMARDs: 6 (20%) versus 3 (10%), *P* = 0.5.

Comparisons between JIA groups according to the type of the disease (oligoarticular, polyarticular, and systemic), regarding cardiovascular risk factors and subclinical markers of atherosclerosis, were not significant (data not shown).

### 3.4. Correlations between IMT, FMD, LVMi, and Traditional Cardiovascular Risk Factors and Inflammatory Markers in the Study Group

In the group of JIA patients, several significant correlations were found between subclinical markers of atherosclerosis, traditional risk factors for CVD, and inflammatory markers. All analyses are presented in Table 5. No associations were observed between subclinical markers of atherosclerosis and total cholesterol, LDL-cholesterol, triglycerides, glucose, and adiponectin levels or between disease duration and onset either. We also found several significant correlations between SDS-BMI and other traditional risk factors: total cholesterol, HDL-cholesterol, HOMA, SBP, and DBP and between inflammation markers: hsCRP and Il-6. Adiponectin level did not correlate with SDS-BMI. 

### 3.5. Independent Relationship between Surrogate Atherosclerosis Markers, Traditional Cardiovascular Risk Factors, and Inflammation Markers

In the multiple regression analysis model taking into account solely the predictive value of traditional risk factors for increased IMT in patients with JIA, SDS-BMI, HOMA, SBP, and DBP were the best predictors, and the model was significant (Table 6, Model 1). When the data were adjusted for inflammatory markers, SBP and DBP together with TNF*α* were the best predictors of the model (Table 6, Model 2). In the model with FMD as the response variable, SDS-BMI and HOMA were the best predictors in Model 1, and in extended inflammatory model SDS-BMI and TNF*α* appeared to be significant predictors (Model 2). For LVMi, we found that SDS-BMI was the only significant predictor in traditional risk factor model (Model 1) and remained significant in extended inflammatory model (Model 2). 

## 4. Discussion

Our main finding is that patients with juvenile chronic arthritis (JIA) had higher body mass, measured as SDS-BMI, although crude values of BMI were similar to those in the control group. Patients with JIA were also characterized by higher systolic and diastolic blood pressure values and increased inflammatory markers. Increased IMT and LVMi and impaired endothelial function, assessed as FMD of brachial arteries, strongly supported the evidence of subclinical changes in cardiovascular system predisposing to early development of clinically symptomatic atherosclerosis. 

Interestingly, 22% of children with JIA met the criteria for overweight or obesity relating to updated growth references for Polish children. This group of children appeared to have a number of increased cardiovascular risk factors typically associated with metabolic syndrome (MS): insulin resistance, dyslipidemia, and increased systolic blood pressure, along with increased inflammatory markers such as hsCRP and IL-6 in comparison to nonobese patients and controls. These alterations were associated with increased ultrasonographic markers for early atherosclerosis. To our knowledge, this is the first report concerning the presence of subclinical atherosclerosis in obese individuals with JIA during growth. 

The prevalence of obesity in children and adolescents increased substantially in the past decades, with current estimates indicating that 31.7% of children and adolescents in the United States are overweight and 16.9% are obese [23]. Recent Polish data report the prevalence of overweight or obesity being 18.7% in boys and 14.1% in girls, aged 7–18 years [17]. Data from the present study show that overweight and obesity rates in children with chronic inflammatory joint disease are higher than those in the population of similar origins. As no further data on obesity in children with JIA are available, a discussion may only remain speculative on the topic. 

Childhood obesity is associated with established risk factors and accelerated atherosclerosis [24, 25]. The prevalence of MS is high among obese children and increases with worsening obesity [26]. The prevalence of metabolic syndrome in patients with JIA, has not been assessed so far. Our studied children, with recognized obesity, clustered metabolic syndrome factors, that is, elevated blood pressure, dyslipidemia, insulin resistance, and, additionally, increased inflammatory markers, relative to JIA nonobese and to controls. Adults with RA are more likely to have MS than non-RA subjects from the same population [27, 28]. The interplay of cytokines, disease activity, use of glucocorticoids, and risk factors clustered in metabolic syndrome in RA is complex. Pathogenic processes driving inflammation in these conditions (RA, MS, and CVD) may involve different pathways and cytokines [29–32]. 

The association between obesity and inflammation in children was first described by Cook et al. who reported much more higher CRP levels in children with excess body mass than in those with normal-weight [33, 34]. Further studies investigating links between CRP, TNF*α*, and IL-6 and pediatric obesity have shown that the proinflammatory state is detectable in obese children, even before other comorbidities of MS are present [35–37]. Results from our study support the view by indicating that obese JIA patients had the highest levels of inflammatory markers, although, unaffected by the disease clinical activity. 

The majority of studies demonstrate that subclinical atherosclerosis, vascular stiffness, and endothelial dysfunction are more prevalent in RA compared to controls [38]. The evaluation of flow mediated dilation (FMD) is now increasingly used for pediatric cardiovascular risk evaluation. Endothelial dysfunction appears to be the first marker of atherosclerosis, as it was revealed in obese children, while no changes in IMT were found [39]. We showed previously the associations between plasma markers of the impaired endothelium, diminished FMD, and increased IMT in obese hypertensive adolescents [20]. In the present study, we demonstrate that children with JIA have impaired endothelial function, with the lowest FMD found in children with coexisting obesity. FMD was independently associated with several traditional cardiovascular risk factors (e.g., BMI) in our patients. In recent-onset RA adult patients, impaired FMD was also reported, and improvement in FMD was noticed together with better disease control [40]. This observation proves the necessity for early detection of early cardiovascular changes that gain importance in primary prevention during growth. 

In adults, increased IMT is an indicator of generalized atherosclerosis and a strong predictor of future cardiovascular events [41] whereas in children it is related to the degree of overweight, chronic inflammation, hypertension, and impaired glucose tolerance [42]. In our study, IMT was increased in JIA children, reaching the highest value in the obese. Furthermore, we noticed a significant correlation with insulin resistance. In adult RA patients, accelerated atherosclerosis appeared a common finding when assessed with use of IMT [43]. Even if in the newly diagnosed RA patients, IMT values were similar to those in controls, a rapid progress of IMT was recorded only in the RA patients during followup [44]. It has been therefore proposed that RA patients should be screened by ultrasonography to identify high-risk individuals requiring more aggressive therapies [45].

In our study, children with JIA had increased indexed left ventricle mass (LVM), and the difference in LVMi between JIA obese and nonobese was also significant. Increased LVM is a recognized predictor of cardiovascular morbidity and mortality [46]. Obesity in childhood and adolescence has also been associated with adversity in cardiac geometry [47–49]. Observations from the Bogalusa Heart Study have shown that change in weight and blood pressure during childhood is predictive of excess LVM in young adults [49]. Crowley et al. indicate associations between secular trends to higher LVMi and increasing BMI, consistently with our findings [50].

The present study highlights the importance of obesity in the development of subclinical atherosclerosis in patients with JIA. The increasing research in this area reveals the complex inflammatory-mediated interactions between adipose tissue, cardiometabolic disorders, and rheumatic diseases. Dysregulation of adipokines, presumably resulting from both obesity and rheumatic diseases, might explain some of the subclinical changes in cardiovascular system as early as that in childhood JIA. The cause of adiposity and its complications in general population, but also in JIA, are a combination of negative nutritional habits, overeating, energy imbalance, and physical inactivity. Therefore, the strategy for therapeutic interventions focused on healthy lifestyle is necessary to obtain adequate modulation of inflammatory response and a reduction of cardiovascular risk at early stage of chronic arthritis. 

There are certain limitations of our study implicating a careful interpretation of the conclusions; that is, the sample size was relatively small limiting power for analysis or some methodological issues. Furthermore, only nonobese controls were included in the analysis; thus, it would be worth examining the differences between obese, otherwise, healthy children and obese JIA patients. Furthermore, we are aware that our patients were taking medications (glucocorticosteroids or anti-TNF*α* therapy) that are known to influence the cardiovascular risk in adults. We did not include detailed drug analysis into the study (dose of corticosteroids or time of the treatment with the above-mentioned drugs), due to a large number of other variables studied, but this presumably would not have altered our results. Obese compared to nonobese JIA patients did not differ significantly in treatment methods and a similar number of patients were corticosteroid users in both groups. Noteworthy, all our analyses were adjusted for steroids use. Despite these limitations, we believe that our data may still contribute to understand some mechanisms of early atherosclerosis and to elucidate the CVD risk in obese JIA patients.

## 5. Conclusions

In conclusion, children and adolescents with JIA demonstrate high overweight and obesity rates. Excess of body weight is associated not only with increased systolic blood pressure, insulin resistance, and dyslipidemia but also with significantly increased levels of inflammatory markers, typical for systemic inflammation in chronic arthritis. Obese JIA patients demonstrate deterioration in cardiovascular system, that is, impaired endothelial function and increased carotid intima-media thickness accompanied by increased left ventricle mass index. This subclinical atherosclerosis depends mainly on obesity-related risk factors; thus, coincidence of the two conditions in childhood may considerably increase CVD risk. Our data show that management of children with JIA, currently focusing on effective treatment and control of the disease activity, should also include individual strategies to maintain appropriate body weight in order to prevent cardiovascular disease in the future. 

## Figures and Tables

**Table 1:** Demographic and clinical characteristics of the juvenile idiopathic arthritis (JIA) patients and the control group.

	JIA patients	Control group
Number of patients	*n* = 58	*n* = 36
Age, years	13.0 (11.0–15.0)	13.4 (12.0–15.0)
Gender		
Boys	26 (45%)	14 (39%)
Girls	32 (55%)	22 (61%)
Height, meter	1.5 (1.4–1.7)	1.6 (1.5–1.7)
Body mass, kg	49.0 (33–65)	51.5 (43–58)
Age at JIA diagnosis, years	9.0 (4.5–12.0)	
Duration of the disease, years	4.0 (2.0–5.5)	
Activity of the disease		
Clinically active	30 (52%)	
Clinically inactive	28 (48%)	
Type of the disease		
Oligoarticular	28 (48%)	
Polyarticular	26 (45%)	
Systemic	4 (7%)	
Current treatment		
Corticosteroids	42 (72%)	
Methotrexate	28 (48%)	
Biologic agents	14 (24%)	
DMARDs	9 (15%)	

Values are the median (interquartile range) or numbers (%).

**Table 2:** Traditional cardiovascular risk factors, selected inflammatory markers, and ultrasonographic evaluation of cardiovascular system in the juvenile idiopathic arthritis (JIA) patients and the control group.

	JIA patients (*n* = 58)	Control group (*n* = 36)	*P* value*
Traditional risk factors			

Age, years	13.0 (11.0–15.0)	13.4 (12–15)	0.37
BMI, kg/m^2^	20.6 (16.8–22.2)	19.4 (17.3–20.6)	0.15
BMI-SDS	0.3 (0.0–0.8)	0.1 (−0.5–0.5)	0.03
SBP, mm Hg	117 (102–127)	112 (103–117)	0.02
DBP, mm Hg	67 (64–75)	64 (57–69)	0.03
Total cholesterol, mmol/L	4 (3.6–4.6)	4 (3.5–4.4)	0.60
LDL-cholesterol, mmol/L	2.1 (1.8–2.5)	2 (1.8–2.4)	0.63
HDL-cholesterol, mmol/L	1.4 (1.2–1.6)	1.4 (1.2–1.7)	0.40
Triglycerides, mmol/L	0.7 (0.6–1)	0.7 (0.6–0.9)	0.74
Glucose, mmol/L	4.8 (4.6–5.1)	4.8 (4.6–5.0)	0.75
Insulin, mU/mL	7.5 (4.1–11.5)	6.6 (4.0–8.8)	0.19
HOMA index	1.5 (0.9–2.4)	1.4 (0.8–1.9)	0.19

Inflammatory markers			

hsCRP, mg/L	1.07 (0.16–3.84)	0.04 (0.01–0.20)	<0.001
Il-6, pg/ml	2.3 (0.5–6.6)	0.2 (0.2–0.8)	<0.001
TNF*α*, pg/ml	2.8 (1.0–32)	0.7 (0.7–0.72)	<0.001
Adiponectin, *μ*g/mL	11.3 (8.0–14.0)	12.5 (8.0–16.0)	0.510

Ultrasonographic studies			

Brachial artery, mm	3.7 (3.3–4.4)	3.6 (3.2–4.2)	0.560
FMD, %	7.8 (5.1–9.1)	11.1 (9.2–12.6)	<0.001
IMT, mm	0.50 (0.44–0.54)	0.42 (0.39–0.45)	<0.001
LVMi, g/m^2.7^	27.6 (24.5–29.5)	22.9 (19.2–28.3)	=0.020

Values are presented as median (interquartile range), SBP: systolic blood pressure, DBP: diastolic blood pressure, HOMA index: index of insulin resistance, FMD: flow mediated dilatation of the right brachial artery, IMT: intima-media thickness of the common carotid arteries, and LVMi: indexed left ventricle mass.

*Mann-Whitney test.

**Table 3:** Traditional cardiovascular risk factors, selected inflammatory markers, and ultrasonographic evaluation of cardiovascular system in nonobese and obese juvenile idiopathic arthritis (JIA) patients.

	JIA patients obese (*n* = 13)	JIA patients nonobese (*n* = 45)	Control group (*n* = 36)	*P* value*
Traditional risk factors				

Age, years	13.0 (7.5–15)	13.0 (11–15.5)	13.4 (12–15)	0.580
BMI, kg/m^2^	24.0 (21–29)^†‡^	19.0 (16–21)	19.4 (17.3–20.6)	<0.001
BMI-SDS	3.0 (1.7–4.2)^†‡^	0.06 (−0.2–0.4)	0.12 (−0.5–0.5)	<0.001
SBP, mm Hg	127 (113–139)^†^	117 (101–124)	112 (103–117)	0.020
DBP, mm Hg	69 (58–80)	67 (64–75)	64 (57–69)	0.130
Total cholesterol, mmol/L	4.7 (4.3–5.0)^†‡^	3.9 (3.6–4.3)	4 (3.6–4.4)	0.010
LDL-cholesterol, mmol/L	2.4 (1.8–2.6)	2.0 (1.7–2.7)	2.0 (1.8–2.4)	0.570
HDL-cholesterol, mmol/l	1.2 (0.9–1.7)	1.5 (1.3–1.6)	1.6 (1.2–1.7)	0.160
Triglycerides, mmol/L	1.3 (0.7–1.5)^‡^	0.7 (0.6–0.9)	0.7 (0.6–0.9)	0.040
Glucose, mmol/L	4.8 (4.5–5.1)	4.8 (4.7–5.1)	4.8 (4.6–5.0)	0.690
Insulin, mU/mL	12.4 (11.0–14.3)^†‡^	6.8 (3.8–8.9)	6.6 (4.0–8.8)	<0.001
HOMA index	2.7 (2.1–3.1)^†‡^	1.5 (0.8–2.0)	1.45 (0.8–1.9)	=0.001

Inflammatory markers				

hsCRP, mg/L	5.3 (1.4–7.4)^†‡^	0.6 (0.1–1.6)^†^	0.04 (0.01–0.2)	<0.001
Il-6, pg/mL	8.6 (4.4–13.2)^†‡^	1.4 (0.4–4.2)^†^	0.2 (0.2–0.8)	<0.001
TNF*α*, pg/mL	3.3 (1–32)^†^	2.6 (1–32)^†^	0.7 (0.7–0.72)	<0.001
Adiponectin, *μ*g/mL	12 (11.5–13.6)	10.5 (6.9–15.6)	12.5 (8.0–16.0)	=0.570

Ultrasonographic studies				

Brachial artery, mm	3.8 (3.8–3.9)	3.6 (3.3–4.2)	3.6 (3.2–4.2)	=0.520
FMD, %	4.8 (2.7–5.7)^†‡^	8.2 (6.5–9.6)^†^	11.1 (9.2–12.6)	<0.001
IMT, mm	0.54 (0.51–0.56)^†‡^	0.48 (0.43–0.53)^†^	0.42 (0.39–0.45)	<0.001
LVMi, g/m^2.7^	30.2 (27–45)^†^	26.8 (21.8–28.5)	22.9 (19.2–28.3)	=0.003

Values are presented as median (interquartile range), obese: group of patients with recognized overweight and obesity, SBP: systolic blood pressure, DBP: diastolic blood pressure, HOMA index: index of insulin resistance, FMD: flow mediated dilatation of the right brachial artery, IMT: intima-media thickness of the common carotid arteries, and LVMi: indexed left ventricle mass.

*ANOVA Kruskal-Wallis test.

^†^ *P* < 0.05 in comparison to the control group in post hoc test.

^‡^ *P* < 0.05 obese versus nonobese JIA groups in post hoc test.

**Table 4:** Traditional cardiovascular risk factors, selected inflammatory markers, and ultrasonographic evaluation of cardiovascular system in clinically active and inactive juvenile idiopathic arthritis (JIA) patients.

	JIA patients clinically active (*n* = 30)	JIA patients clinically inactive (*n* = 28)	Control group (*n* = 36)	*P* value*
Traditional risk factors				

Age, years	12.5 (9.5–15.0)	14.0 (11.0–16.0)	13.4 (12.0–15.0)	0.24
BMI, kg/m^2^	20.2 (18.3–22.2)	20 (16.6–21.7)	19.4 (17.3–20.6)	0.19
BMI-SDS	1.1 (0.1–1.7)^†^	0.1 (−0.1–0.5)	0.1 (−0.5–0.5)	0.01
SBP, mmHg	116 (99–127)	121 (103–126)^†^	112 (103–117)	0.03
DBP, mmHg	68 (55–71)	65 (57–69)	64 (57–69)	0.20
Total cholesterol, mmol/L	4.2 (3.7–4.7)	3.9 (3.5–4.4)	4 (3.5–4.4)	0.48
LDL-cholesterol, mmol/L	2.2 (1.8–2.5)	2.0 (1.8–2.5)	2.0 (1.8–2.4)	0.71
HDL-cholesterol, mmol/L	1.4 (1.0–1.5)	1.3 (1.2–1.6)	1.4 (1.1–1.6)	0.51
Triglycerides, mmol/L	0.7 (0.6–1.2)	0.8 (0.5–0.9)	0.7 (0.6–0.9)	0.52
Glucose, mmol/L	4.8 (4.6–5.0)	5.0 (4.8–5.1)	4.8 (4.6–5.0)	0.21
Insulin, mU/mL	7.1 (4.8–13.3)	7.9 (3.8–10.5)	6.6 (4.0–8.8)	0.30
HOMA index	1.5 (1.0–2.7)	1.7 (0.8–2.4)	1.4 (0.8–1.9)	0.28

Inflammatory markers				

hsCRP, mg/L	1.2 (0.3–4.9)^†^	0.8 (0.1–2.7)^†^	0.04 (0.01–0.2)	<0.001
Il-6, pg/mL	3.6 (1.1–7.4)^†^	1.4 (0.4–6.4)^†^	0.2 (0.2–0.8)	<0.001
TNF*α*, pg/mL	2.5 (0.8–17.9)^†^	3 (1.3–32)^†^	0.7 (0.7–0.72)	<0.001
Adiponectin, *μ*g/mL	10.9 (8.0–13.9)	11.0 (8.0–14.0)	12.5 (8.0–16.0)	=0.640

Ultrasonographic studies				

Brachial artery, mm	3.7 (3.3–3.9)	3.8 (3.4–4.5)	3.6 (3.2–4.2)	=0.230
FMD, %	7.8 (6.2–9.6)^†^	8.1 (2.7–8.9)^†^	11.1 (9.2–12.6)	<0.001
IMT, mm	0.49 (0.43–0.51)^†^	0.53 (0.44–0.56)^†^	0.42 (0.39–0.45)	<0.001
LVMi, g/m^2.7^	27.6 (25.1–29.9)^†^	27.6 (18.8–29.1)	22.9 (19.2–28.3)	=0.040

Values are presented as median (interquartile range), SBP: systolic blood pressure, DBP: diastolic blood pressure, HOMA index: index of insulin resistance, FMD: flow mediated dilatation of the right brachial artery, IMT: intima-media thickness of the common carotid arteries, and LVMi: indexed left ventricle mass.

*ANOVA Kruskal-Wallis test.

^†^ *P* < 0.05 in comparison to the control group in post-hoc test.

^‡^ *P* < 0.05 clinically active versus inactive groups in post-hoc test.

**Table 5:** Correlations (Spearman correlation coefficient—rho) between IMT, FMD, LVMi, SDS-BMI, and traditional cardiovascular risk factors and inflammatory markers in the study group.

	IMT	FMD	LVMi	SDS-BMI
BMI	rho = 0.51, *P* < 0.001	rho = −0.30, *P* = 0.001	rho = 0.38, *P* = 0.001	rho = 0.89, *P* < 0.001
SDS-BMI	rho = 0.45, *P* < 0.001	rho = −0.30, *P* < 0.001	rho = 0.43, *P* < 0.001	—
HOMA	rho = 0.29, *P* < 0.001	rho = −0.34, *P* < 0.001	rho = 0.32, *P* = 0.008	rho = 0.41, *P* = 0.001
SBP	rho = 0.54, *P* < 0.001	rho = −0.20, *P* = 0.010	rho = 0.32, *P* = 0.006	rho = 0.47, *P* < 0.001
DBP	rho = 0.21, *P* = 0.050	rho = −0.14, *P* = 0.160	rho = 0.04, *P* = 0.700	rho = 0.38, *P* = 0.002
TC	rho = 0.03, *P* = 0.700	rho = −0.10, *P* = 0.300	rho = 0.09, *P* = 0.400	rho = 0.27, *P* = 0.03
LDL	rho = 0.01, *P* = 0.800	rho = −0.13, *P* = 0.190	rho = 0.06, *P* = 0.600	rho = 0.05, *P* = 0.6
HDL	rho = −0.36, *P* < 0.001	rho = 0.21, *P* = 0.030	rho = −0.25, *P* = 0.030	rho = −0.35, *P* = 0.01
TG	rho = 0.14, *P* = 0.170	rho = −0.10, *P* = 0.300	rho = 0.23, *P* = 0.190	rho = 0.13, *P* = 0.18
hsCRP	rho = 0.38, *P* < 0.001	rho = −0.3, *P* = 0.005	rho = 0.31, *P* = 0.016	rho = 0.46, *P* < 0.001
IL-6	rho = 0.35, *P* = 0.001	rho = −0.34, *P* = 0.001	rho = 0.08, *P* = 0.400	rho = 0.36, *P* = 0.006
TNF*α*	rho = 0.36, *P* < 0.001	rho = −0.36; *P* < 0.001	rho = 0.33, *P* = 0.009	rho = 0.02, *P* = 0.8

**Table 6:** Multiple linear regression analyses for IMT, FMD, and LVMi as dependent variables.

	Model 1			Model 2		
Dependent variable	Independent variable	*β*	*P*	Independent variable	*β*	*P*

	Age	0.09	0.460	Age	0.20	0.17
	SDS-BMI	0.25	0.010	SDS-BMI	0.21	0.10
	HOMA	0.18	0.050	HOMA	0.17	0.12
IMT	HDL	−0.16	0.070	HDL	−0.11	0.25
	SBP	0.42	0.001	SBP	0.37	0.02
	DBP	0.27	0.020	DBP	0.26	0.05
				Il-6	0.04	0.65
				TNF*α*	0.20	0.04
				hsCRP	0.02	0.79
	*R* ^2^ = 0.42, *P* < 0.001			*R* ^2^ = 0.47, *P* < 0.001		

	Age	−0.04	0.75	Age	−0.20	0.21
	SDS-BMI	−0.22	0.06	SDS-BMI	−0.29	0.04
	HOMA	−0.26	0.01	HOMA	−0.19	0.13
	HDL	0.08	0.39	HDL	−0.03	0.75
FMD	SBP	−0.01	0.80	SBP	0.1	0.54
				Il-6	−0.16	0.16
				TNF*α*	−0.24	0.04
				hsCRP	−0.04	0.71
	*R* ^2^ = 0.22, *P* < 0.001			*R* ^2^ = 0.30, *P* < 0.001		

	Age	−0.01	0.880	Age	0.02	0.890
	SDS-BMI	0.43	0.003	SDS-BMI	0.53	0.004
	HOMA	0.14	0.270	HOMA	0.07	0.620
LVMi	HDL	−0.09	0.400	HDL	−0.01	0.880
	SBP	0.05	0.720	SBP	0.02	0.860
				TNF*α*	0.20	0.100
				hsCRP	−0.03	0.980
	*R* ^2^ = 0.36, *P* < 0.001			*R* ^2^ = 0.41, *P* < 0.001		

Multiple linear regression analyses for IMT, FMD, and LVMi as dependent variables in models of traditional atherosclerosis risk factors (Model 1) and extended model with the conjunction with the inflammatory markers (Model 2), SBP: systolic blood pressure, DBP: diastolic blood pressure, and HOMA: insulin resistance index.

## Abbreviations

BMI: Body mass index
CRP: C-reactive protein
CV: Cardiovascular
CVD: Cardiovascular disease
FMD: Flow mediated dilation
ELISA: Enzyme-linked immunosorbent assay
IMT: Intima-media thickness
LVMi: Left ventricle mass index
sICAM-1: Soluble intercellular adhesion molecule-1
TNF: Tumor necrosis factor.
